# *Kytococcus* Species Infections in Humans—A Narrative Review

**DOI:** 10.3390/microorganisms13051072

**Published:** 2025-05-04

**Authors:** Petros Ioannou, Eleni Kampanieri, Stergos Koukias, Stella Baliou, Andreas G. Tsantes, Diamantis Kofteridis

**Affiliations:** 1 School of Medicine, University of Crete, 71003 Heraklion, Greece; 2 Internal Medicine Department, University Hospital of Heraklion, 71110 Heraklion, Greece; 3 Internal Medicine Department, General Hospital of Chania, 73300 Chania, Greece; 4 Internal Medicine Department, Venizeleio General Hospital, 71409 Heraklion, Greece; 5 Laboratory of Hematology and Blood Bank Unit, “Attikon” University Hospital, School of Medicine, National and Kapodistrian University of Athens, 12462 Athens, Greece

**Keywords:** *Kytococcus*, bacteremia, infection, infective endocarditis, respiratory tract infection, osteomyelitis

## Abstract

*Kytococcus* belongs to the family Kytococcaceae, in the order Micrococcales, in the class Actinomycetes, and the phylum Actinomycetota. *Kytococcus* are aerobic, Gram-positive, non-spore forming bacteria that form coccoid, non-motile, non-encapsulated cells, and their colonies on agar have a yellow color. Infections by these species are increasingly identified nowadays. This narrative review aimed to present all available cases of *Kytococcus* spp. infections in humans, emphasizing data on the epidemiology, antimicrobial resistance, antimicrobial treatment, and mortality. A narrative review based on a literature search of the PubMed/MedLine and Scopus databases was performed. Results: In total, 26 articles providing data on 30 patients with *Kytococcus* spp. infections were included in this analysis. The median age was 59.5 years, while 56.7% were male. The presence of a prosthetic cardiac valve was the main predisposing factor in 36.7% of patients (100% among those with infective endocarditis), while immunosuppression due to underlying hematological malignancy under chemotherapy was the second most common. Bacteremia was the most common type of infection, with infective endocarditis being the most common subtype in this infection type, while respiratory tract infections and osteoarticular infections were also relatively common. *K. schroeteri* was the most commonly identified species. Microbial identification required the use of advanced molecular techniques such as 16s rRNA sequencing in most cases. *Kytotoccus* spp. was resistant to all beta-lactams with the exception of carbapenems and macrolides. The most commonly used antimicrobials were vancomycin and rifampicin. Mortality was significant (30%). Due to the potential of *Kytotoccus* spp. to cause infective endocarditis in patients with prosthetic cardiac valves and respiratory tract infections with concomitant bacteremia in patients with hematological malignancy under chemotherapy along with the difficulties in pathogen identification, clinicians and laboratory personnel should consider this pathogen in the differential diagnosis in patients with typical predisposing factors and clinical presentation, especially when traditional microbiological techniques are used for pathogen identification.

## 1. Introduction

The genus *Micrococcus* consists of Gram-positive, catalase-positive cocci, belongs to the group of actinomycete, and was first described more than a century ago [1,2]. Phylogenetic and chemotaxonomic analyses have led to several changes in the classification of bacteria from the genus *Microoccus*. The genus *Kytococcus* was first distinguished from the genus *Micrococcus* in 1995 [1]. *Kytococcus* belongs to the family Kytococcaceae, in the order Micrococcales, in the class Actinomycetes, and the phylum Actinomycetota [1,3]. *Kytococcus* are aerobic, Gram-positive, non-spore forming bacteria that form coccoid, non-motile, non-encapsulated cells, and their colonies on agar have a yellow color [3,4]. Its peptidoglycan is of the A4α type and contains L-lysine as the diamino acid. It does not contain teichoic acids or mycolic acids, and the genomic G+C content is 68–73% [3]. The genus includes three species, namely, *Kyotococcus aerolatus, Kyotococcus schroeteri*, and *Kyotococcus sedentarius*, while the NCBI taxonomy browser reports several unclassified and uncultured variants [5].

Infections by *Kytococcus* species are quite uncommon and are usually reported in the form of case reports with a literature review. Thus, data on the epidemiology, type of infection, diagnostic modalities, antimicrobial susceptibility, treatment, and outcomes remain scarce. To this end, the present study aimed to comprehensively review all the available information of human infections caused by *Kytococcus* spp. in the literature and to assess the clinical data, microbiology, treatment, and outcomes by performing individual patient data analysis from already published studies.

## 2. Materials and Methods

### 2.1. Search Strategy and Inclusion and Exclusion Criteria

This narrative review aimed to collect and present all of the published data on *Kytococcus* species infections in humans after performing individual patient data analysis from already published studies. The primary aim of the study was to present data on the patients’ epidemiology and mortality. The secondary aims were to collect data about the specific infection site, the clinical presentation, the microbiological features, and the treatment provided. For this review, the PubMed/Medline and Scopus databases were searched until 27 February 2025. Data were extracted using a predefined template. The following keywords were applied for the search strategy: “*Kytococcus*” AND “infection”. Studies providing original data, such as case series, case reports, and cohort studies providing information into the epidemiology and clinical outcomes of *Kytococcus* spp. infections in humans, were included. Studies in language other than English, reviews, and systematic reviews were excluded. Studies involving animals, articles without full-text access, and those lacking information on the patients’ mortality and epidemiology were also excluded from the analysis. Additionally, cases of colonization by *Kytococcus* species were excluded from the analysis, based on the clinical course depicted and the study authors’ consideration as to whether the species were pathogens or colonizers. The references of all included articles were examined to identify any studies potentially missed in the initial search.

### 2.2. Data Extraction and Definitions

The data extracted from each included study were publication year, article type, country of origin, patient demographics (age, gender), relevant medical history, details of infection, and key clinical characteristics such as specific infection site and complications as well as microbiological characteristics such as identified pathogen, antibiotic susceptibilities, and finally, treatment used and outcome (survival or mortality). The relationship between mortality and the initial infection was documented according to each study’s authors. This information from already published studies was transferred to a predefined datasheet and processed further by performing individual patient data analysis.

### 2.3. Statistical Analysis

Data are presented as numbers (%) for the categorical variables and the median (interquartile range, IQR) for continuous variables. Continuous variables were compared using the Mann–Whitney U-test for non-normally distributed variables or the *t*-test for normally distributed variables. All tests were two-tailed, and a *p*-value equal to or lower than 0.05 was considered significant. A univariate linear regression analysis including all patients, irrespective of age, was conducted to identify factors associated with all-cause mortality. Statistics were calculated with GraphPad Prism 6.0 (GraphPad Software, Inc., San Diego, CA, USA). A multivariate linear regression analysis was conducted in order to evaluate the effect of factors previously identified in the univariate analysis model to be associated with all-cause mortality with a *p* < 0.05. Multivariate analysis was performed using the SPSS version 23.0 (IBM Corp., Armonk, NY, USA).

## 3. Results

### 3.1. Included Studies’ Characteristics

**Figure 1:**
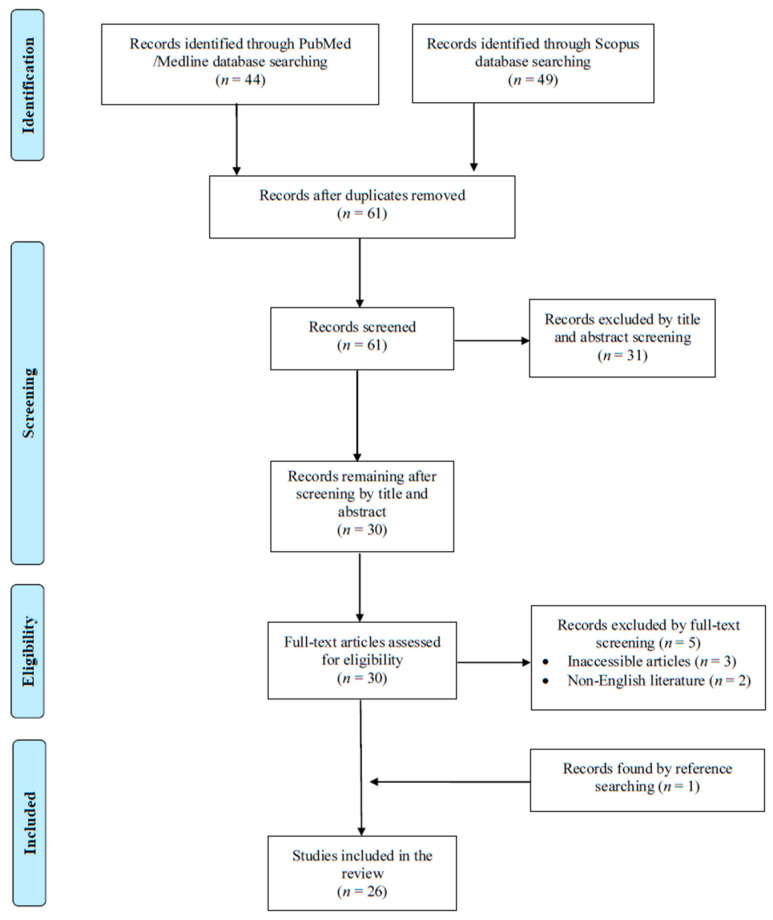
Trial flow of this narrative review.

A total of 93 articles were screened from the PubMed and Scopus databases. Eventually, after duplicate removal, record screening, and applying the snowball procedure, only 26 articles met the inclusion criteria and were selected for analysis [6,7,8,9,10,11,12,13,14,15,16,17,18,19,20,21,22,23,24,25,26,27,28,29,30,31]. These studies presented data on 30 patients. A flow diagram of the selection process is illustrated in Figure 1. Among the included cases, 16 occurred in Europe (61.6%), 5 in North and South America (19.2%), and 5 in Asia (19.2%). Among the 26 included articles, 23 (88.5%) were case reports. Table 1 shows the characteristics of the included studies in the present review.

### 3.2. Epidemiology of Kytococcus spp. Infections

The median age of patients with *Kytococcus* spp. infections was 59.5 years, with a range of 0 to 83 years, while 56.7% (17 patients) were male. Regarding the patients’ medical history and predisposing risk factors, 11 out of 30 patients (36.7%) had a prosthetic cardiac valve, 8 (26.7%) had immunosuppression due to hematological malignancy undergoing treatment, 2 patients (6.7%) had a history of recent surgery within the previous three months, 1 (3.3%) had end stage renal disease on dialysis, 1 (3.3%) previously had infective endocarditis, and 1 (3.3%) had rheumatic fever. The demographic and clinical characteristics of patients with infections by *Kytococcus* spp. are shown in Table 2.

### 3.3. Microbiology and Antimicrobial Resistance of Kytococcus spp. Infections

*Kytococcus* spp. was isolated from blood cultures in 20 patients (66.7%), from bronchoalveolar fluid in 4 (13.3%), from tissue cultures in 4 (13.3%), from valve culture in 2 (6.7%), from bone culture in 2 (6.7%), and from joint fluid, peritoneal fluid, extracted port catheter, pus, and sputum in 1 (3.3%) each. *K. schroeteri* was the identified species in 25 patients (83.3%), *K. sedentarius* was identified in 4 (13.3%), and in 1 patient (3.3%) the species was not reported. In 17 cases (56.7%), identification was performed with 16s rRNA sequencing. In 10 cases (33.3%), identification was performed with matrix-assisted laser desorption/ionization time of flight mass spectrometry (MALID-TOF MS). In one case (3.3%), identification was performed with VITEK2, and in one case (3.3%), the identification was performed with phenotypic and molecular methods. The means of pathogen identification was not mentioned in three cases (10%). The antimicrobial resistance of *Kytococcus* spp. is shown in Table 3. In 2 out of 22 patients (6.7%), the infection was polymicrobial.

### 3.4. Clinical Presentation of Kytococcus spp. Infections

The most common type of *Kytococcus* spp. infections were those of the bloodstream in 20 patients (66.7%). Infective endocarditis was diagnosed in 12 patients (40%). Other infections were lower respiratory tract infections in six patients (20%), osteoarticular infections in five (16.7%), skin and soft tissue infections in two (6.7%), central nervous system infection in one (3.3%), vascular graft infection in one (3.3%), and peritoneal dialysis-associated infection in one (3.3%). The symptoms’ durations ranged from 0 (acute onset) to more than a year.

### 3.5. Treatment and Outcome of Kytococcus Infections

The treatment of patients with *Kytococcus* spp. infections in shown in detail in Table 1 and is also summarized in Table 2. Based on the available data, vancomycin was the most frequently administered antimicrobial used in 20 out of 28 patients with available data (71.4%), followed by rifampicin (used in combination with other antimicrobials) in 11 patients (39.3%), aminoglycosides in 7 (25%), quinolones in 6 (21.4%), carbapenems in 4 (14.3%), an aminopenicillin with an inhibitor in 2 (7.1%), cephalosporin in 2 (7.1%), daptomycin in 2 (7.1%), linezolid in 2 (7.1%), tetracycline in 1 (3.6%), trimethoprim with sulfamethoxazole in 1 (3.6%), anti-staphylococcal penicillin in 1 (3.6%), and teicoplanin in 1 (3.6%). Surgical interventions were applied in combination with antimicrobial treatment in 16 out of 30 patients (53.3%). The median treatment duration for survivors was 6 weeks. The overall mortality rate was estimated at 30% (9 out of 30 patients), with the mortality directly associated with the *Kytococcys* spp. infection being 26.7% (8 patients).

### 3.6. Bacteremia Due to Kytococcus

Bacteremia was diagnosed in 20 patients (66.7%). Among them, 12 (60%) were male and the median age was 51.5 years. Among these patients, six (30%) had immunosuppression due to hematological malignancy that was under treatment. Infective endocarditis was diagnosed in 11 patients (55%) with bacteremia while a lower respiratory tract infection was present in 4 (20%), and central nervous infection was present in 1 (5%). Only in one patient with an infection of the lower respiratory tract was the infection polymicrobial. Fever was present in 16 patients (80%), and sepsis was present in 11 (55%). Vancomycin, rifampicin, and aminogylcosides were the most commonly used antimicrobials. The overall mortality was 35% (7 patients).

### 3.7. Infective Endocarditis Due to Kytococcus

Infective endocarditis was diagnosed in 11 patients. The median age in this patient group was 64 years, and seven patients were male (63.6%). A prosthetic cardiac valve was present in all patients (100%), and was bioprosthetic in seven out of ten patients with available data (70%) and metallic in three (30%). Previous infective endocarditis was noted in the history of one patient (9.1%), rheumatic fever in one (9.1%), and one patient (9.1%) had a history of recent surgery in the three months before presentation. All patients had bacteremia, and the diagnosis was made with blood cultures in all patients, valve culture in three patients (27.3%), polymerase chain reaction (PCR) in the valve in two patients (18.2%), valve histology in one patient (9.1%), transesophageal echocardiography in nine patients (81.8%), and transthoracic echocardiography in two (18.2%). The infected intracardiac site was the aortic valve in nine patients (81.8%), and the mitral valve in two (18.2%). Fever was present in eight patients (72.7%), and sepsis was present in five (45.5%). Embolic phenomena developed in three patients (27.3%), and heart failure developed in one patient (9.1%). A paravalvular abscess was noted in six patients (54.5%). The median treatment duration was 6 weeks. The most commonly used antimicrobial agents were vancomycin in ten patients (90.9%), rifampicin in eight (72.7%), and aminoglycosides in seven (63.6%). The overall mortality was 9.1%, with only one patient dying due to the episode of *Kytococcus* infective endocarditis.

### 3.8. Lower Respiratory Tract Infection Due to Kytococcus

A lower respiratory tract infection was diagnosed in seven patients (23.3%). Among them, four (57.1%) were male, and the median age was 52 years. Among patients with this infection, six (85.7%) had immunosuppression due to hematological malignancy that was under treatment, and five (71.4%) had bacteremia along with the respiratory tract infection. One patient with hematological malignancy had a skin and soft tissue infection that was confirmed to be due to *Kytococcus*; at the same time, he had a respiratory tract infection. Only in one patient was the infection polymicrobial. Fever was present in six patients (85.7%), and sepsis in two (28.6%). Vancomycin and cephalosporins were the most commonly used antimicrobials. The overall mortality was 85.7% (six patients).

### 3.9. Osteoarticular Infection Due to Kytococcus

An osteoarticular infection was diagnosed in five patients (16.7%). Among them, one (20%) was male, and the median age was 71 years. Among patients with this infection, one (20%) had surgery in the three months preceding the diagnosis. Fever was present in one patient (20%). Quinolone was the most commonly used antimicrobial for treatment, and surgical management was performed in four patients (80%). No patient died.

### 3.10. Characteristics of Patients with Kytococcus spp. with Regard to Survival

Table 4 shows a comparison of the characteristics of patients with *Kytococcus* spp. who lived with those who died. Patients who died were more likely to have had immunosuppression due to hematological malignancy on chemotherapy, more likely to have had an infection of the lower respiratory tract, and were less likely to have had treatment with rifampicin or have had surgical treatment along with antimicrobials. These results more likely reflect the high mortality rate of respiratory tract infections in patients with immunosuppression due to hematological malignancy, the use of rifampicin in patients with infective endocarditis and vascular graft infections, and the high rate of surgical intervention in patients with infective endocarditis and osteoarticular infections that had a small mortality rate.

To identify factors independently associated with overall mortality, we first performed a linear regression of gender, age, presence of a prosthetic valve, type of infection, presence of fever or sepsis, and antimicrobial treatment provided. Infection of the lower respiratory tract and presence of immunosuppression were identified as being positively associated with mortality (*p* < 0.0001 and *p* = 0.0006, respectively). However, a multivariate logistic regression analysis did not identify any factor to be independently associated with mortality. The results of the multivariate regression can be seen in Table 5.

## 4. Discussion

This review summarized the clinical and microbiological characteristics of human infections by *Kytococcus* spp. by evaluating all published studies providing relevant data in the literature and by presenting data from individual patient data analysis from these already published studies. The most common types of infections were bacteremia, infective endocarditis, respiratory tract infections, and osteoarticular infections. Antimicrobial resistance to first-line beta-lactams was almost universal while vancomycin, daptomycin, linezolid, and cabapenems were active in almost all cases, and vancomycin was the most commonly used antimicrobial for treating infections by these pathogens. Mortality from infections by *Kytococcus* spp. was high, especially for immunosuppressed patients and those with respiratory tract infections.

*Kytococcus* species were dissected from the genus *Micrococcus* after the work of Stackebrandt et al. in 1995 [1]. Ever since, several reports of infections have been reported with identification requiring advanced molecular techniques such as 16s rRNA sequencing or MALDI-TOF MS. Indeed, there seem to be several difficulties with the correct identification of this microorganism. For example, a recent study in Japan revealed that cases of human infection by *Kytococcus* spp. may have been missed due to misidentification as *Micrococcus* spp. [32]. Moreover, due to the fact that *Kytoococcus* spp. can represent human skin microbiota, further difficulties in its identification may also occur since specimens could be discarded as being considered to have been contaminated [27]. Thus, cases of *Kytococcus* spp. human infection could be underreported. In the present review, 16s rRNA sequencing was the most common means of pathogen identification, and was used in more than half the cases, followed by MALDI-TOF MS. These techniques have led to a revolution in microbiology, facilitating the identification of uncommon microorganisms that would be very difficult to identify based on the use of traditional biochemical and morphological pathogen characteristics [33,34,35]. To this end, only one study included in the present review performed identification solely based on the biochemical and morphological characteristics.

The most common conditions in the past medical history of patients suffering an infection by *Kytococcus* spp. were the presence of a prosthetic cardiac valve and immunosuppression, which was due to hematological malignancy that was under treatment in all immunosuppressed patients. The presence of a prosthetic cardiac valve was clearly a predisposing factor for the patients with infective endocarditis, with all patients with this infection having a prosthetic cardiac valve. This is reasonable since the presence of a prosthetic cardiac valve is a well-known factor for the development of infective endocarditis [36,37,38]. However, the fact that all patients with infective endocarditis had a prosthetic cardiac valve is intriguing. This could be associated with the fact that *Kytococcus* spp. is part of the skin microbiome, thus increasing the likelihood that a short episode of bacteremia due to a brief duration of lysis of the epithelial barrier due to trauma or other reasons could lead to inoculation of the prosthetic valve and the development of infective endocarditis. On the other hand, given that the pathogenicity of this microorganism is not clear, it could be reasonable to think that the predilection that *Kytococcus* spp. shows for prosthetic cardiac valves could have a pathophysiological basis. Thus, it could be that this pathogen produces specific substances that allow it to more easily adhere to the prosthetic material of prosthetic cardiac valves, leading to infective endocarditis. However, this has to be shown in studies directly evaluating the pathogenicity of this species.

Immunosuppression due to treatment for hematological malignancy was the second most common condition identified in patients with infection due to *Kytococcus* spp. These patients were more likely to have a respiratory tract infection and bacteremia, while the outcome was commonly fatal. On the other hand, patients with immunosuppression are well-known to be more susceptible to infection and are also more likely to suffer more severe complications, such as sepsis, if infected [39,40]. Since the field of infectious diseases has changed a lot in the last years, and not all immunosuppressed patients are considered identical, it is of utmost importance to understand the exact mechanism leading to immunosuppression and to understand the ‘net state of immunosuppression’ for each particular patient [39]. Patients with hematological malignancies are at a particularly high risk for infections due to the neutropenia that is common in patients on myeloablative chemotherapy or even due to the disease, the hypogammaglobulinemia that is often seen in these patients, the mucosal damage due to therapy, T-cell dysfunction, and the common use of intravascular devices [41,42]. Despite the fact that these patients with such immunosuppression usually had bacteremia and frequently had sepsis, it is unclear as to why they are more likely to have a respiratory tract infection, and since there are scarce data available regarding its pathophysiology, little can be said regarding the route of infection. Future studies should focus on the exact pathogenetic mechanisms leading to these infections in immunosuppressed individuals.

Among the different clinical presentations, bacteremia was the most common presentation and was associated with infective endocarditis in the majority of cases, and second, respiratory tract infections. However, given the rarity of the pathogen, the possible underreporting in the literature, the problems related to the identification of the pathogen, and the possibility of rejecting the diagnosis of bacteremia by discarding a blood culture as a contaminant, it could be that cases of bacteremia without a respiratory tract infection or infective endocarditis, especially in patients with hematological malignancy that have indwelling devices and are under chemotherapy, could have been missed. Other infections noted in the present narrative review include osteoarticular infections, peritoneal dialysis-associated peritonitis, skin and soft tissue infections, and vascular graft infections. These infections are commonly seen with bacteria that are commensals of the skin such as staphylococci [43,44,45,46,47,48]. *Kytococcus* spp. have been also identified in other conditions that do not constitute invasive human infections such as pitted keratolysis [49]. For example, in a case report from a Greek center, clinical specimens from an adolescent with pitted keratolysis revealed the growth of several microorganisms, with two species of *Kytococcus* being among them [49]. Such studies were excluded from the present review, since they do not constitute deep seated infections, while underreporting would have probably been extensive.

Studies regarding the antimicrobial susceptibility of *Kytococcus* spp. are scarce, and studies regarding the genes and the molecular mechanisms underlying this resistance are even scarcer. In a recent study analyzing a *Kytococcus sedentarius* strain isolated from a dehumidifier in a university, several genes associated with the development of antimicrobial resistance were identified [50]. For example, that strain was resistant to ciprofloxacin, gentamicin, and erythromycin. Notably, in that study, the presence of genes encoding multi-drug-resistance pumps was noted, however, the resistance to erythromycin could not be explained based on those genes, implying the presence of other genes coding for proteins and mechanisms associated with resistance to that antimicrobial [50]. In the present study, antimicrobial resistance to macrolides, penicillin, oxacillin, and cephalosporins was higher than 90%. Antimicrobial resistance to vancomycin, linezolid, quinolones, daptomycin, tetracyclines, rifampicin, and carbapenems was low, thus these antimicrobials could be used to treat these infections. Indeed, in most of the studies included in the present review, vancomycin and rifampicin were the two most commonly used antimicrobials, with rifampicin being used as an adjunct antimicrobial mostly in patients with infective endocarditis. Even though this review cannot provide strong recommendations given the rarity of the infection and the very few reports included in the present analysis, appropriate empirical treatment is of paramount importance, especially in severe infections, as in immunosuppressed patients. However, given the inability for international scientific societies to provide guidelines for treating infections by these microorganisms, it is reasonable to consider vancomycin, daptomycin, tetracyclines, quinolones, and linezolid for treatment.

Notably, the treatment duration of the patients included in the present analysis was quite long, and this could be associated with the diagnosis of infective endocarditis in many of them. Moreover, the duration of treatment herein was calculated only for patients who survived, and since patients with infective endocarditis had very low mortality, the duration of their treatment contributed significantly to the duration of treatment of all survivors. More studies are warranted to identify the optimal treatment, and even more so, the optimal duration of treatment for these patients.

The mortality of *Kytococcus* infections was high, with about 30% of patients dying from the infection. Importantly, 67% of patients who died had a hematological malignancy under treatment, 78% of patients who died had bacteremia, and 67% had a respiratory tract infection. These factors were among those that were different in those who died versus those who survived in a statistically significant manner. Moreover, patients who survived were given rifampicin more frequently and were also treated with surgery more frequently; both of these factors can be associated with the treatment of infective endocarditis that was more frequent in those who survived. The discrepancy in the mortality of infective endocarditis, which is traditionally considered a more deadly infection, and that of patients with respiratory tract infection with bacteremia, mostly seen in patients with hematological malignancy, could be partly associated with the particularities noted in patients with underlying immunosuppression and their higher likelihood for worse clinical outcomes in the case of an infection [51]. Further studies are needed to better understand the differences in the clinical syndromes of *Kytococcus* spp. infections.

This study had some limitations. Firstly, the published studies may not have adequately represented the infections by *Kytococcus* spp. due to problems associated with the identification, publication bias, or other reasons. Moreover, some studies may have been missed during the screening process. Additionally, the analysis herein was based exclusively on case reports, which depend on highly accurate record-keeping. Thus, this review only presented findings from studies that provided complete data for the parameters that were evaluated, especially for mortality and epidemiology. Moreover, this review identified only a small number of studies providing data for only a small number of patients, thus limiting the credibility of the conclusions of the study.

## 5. Conclusions

The present review provides important information regarding the epidemiology, clinical presentation, antimicrobial resistance, and treatment and outcomes of *Kytococcus* spp. infections in humans. *K. schroeteri* was the most commonly identified species, while the most common infections were those of the bloodstream, infective endocarditis, and respiratory tract infections. *Kytococcus* was resistant to all beta-lactams with the exception of carbapenems and macrolides, while antimicrobial resistance to vancomycin, daptomycin, linezolid, and tetracyclines was minimal. Vancomycin was the most commonly used for treating infections by these pathogens. Mortality was significant, but mostly for patients with hematological malignancy under treatment that had a respiratory tract infection along with bacteremia. *Kytococcus* spp. infections should be considered in patients with a prosthetic cardiac valve suffering infective endocarditis, or in patients with hematological malignancy and a respiratory tract infection with bacteremia when Gram-positive cocci are isolated, especially when their features resemble those of *Micrococcus*, even though advanced molecular techniques such as MALDI-TOF MS or 16s rRNA sequencing will probably be needed for the correctly identification of this pathogen.

## Figures and Tables

**Table 1:** Characteristics of all of the included studies.

Author, Year	Number of Patients	Gender	Age (Years)	Site of Infection (%)	Treatment (%)	Mortality (%)
Becker et al., 2003 [6]	1	Female	34	Heart, BSI	Vancomycin Aminoglycoside Rifampicin	0
Levenga et al., 2004 [7]	1	Male	55	LRTI	Cephalosporin Vancomycin	1
Le Brun et al., 2005 [8]	1	Male	73	Heart, BSI	Vancomycin Aminoglycoside Rifampicin Teicoplanin	0
Mohammedi et al., 2005 [9]	1	Female	71	LRTI, BSI	Cephalosporin Quinolone	1
Mnif et al., 2006 [10]	1	Female	49	Heart, BSI	Pristinamycin Vancomycin Rifampicin	0
Renvoise et al., 2008 [11]	1	Male	70	Heart, BSI	Vancomycin Aminoglycoside	0
Aepinus et al., 2008 [12]	1	Female	0	Heart, BSI	Vancomycin Quinolone Aminoglycoside Rifampicin	0
Jourdain et al., 2009 [13]	1	Male	1	Central nervous system	Vancomycin Rifampicin	0
Yousri et al., 2010 [14]	1	Male	64	Heart, BSI	Vancomycin Aminoglycoside Rifampicin	0
Jacquier et al., 2010 [15]	1	Female	50	Osteoarticular infection	Quinolone Rifampicin	0
Hodiamont et al., 2010 [16]	2	2 males	40, 52	LRTI 2 (100), BSI 1 (50)	NR 1 (50) Vancomycin 1 (50)	2 (100)
Chaudhary et al., 2010 [17]	1	Female	66	Peritoneal dialysis peritonitis	Vancomycin	0
Nagler et al., 2011 [18]	1	Male	68	LRTI	NR	1
Dainese et al., 2012 [19]	1	Male	67	Vascular graft infection	Aminopenicillin and inhibitor Vancomycin Rifampicin	0
Liu et al., 2011 [20]	1	Male	53	Heart, BSI	Daptomycin	0
Blennow et al., 2012 [21]	1	Female	43	LRTI, BSI	Linezolid Carbapenem Vancomycin Trimethoprim-sulfamethoxazole	0
Chan et al., 2012 [22]	1	Male	45	Osteoarticular infection	Tetracycline	0
Amaraneni et al., 2015 [23]	1	Female	50	LRTI, BSI	Vancomycin	1
DeMartini et al., 2016 [24]	1	Male	17	BSI	Carbapenem Vancomycin	1
Bayraktar et al., 2018 [25]	1	Male	3	BSI	Vancomycin	1
Shah et al., 2019 [26]	1	Female	80	Osteoarticular infection	Daptomycin	0
Bagelman et al., 2021 [27]	1	Female	0.75	BSI	Carbapenem Vancomycin	0
Lim et al., 2021 [28]	1	Female	79	Osteoarticular infection	Quinolone	0
Zellner et al., 2023 [29]	2	1 female, 1 male	66, 71	Osteoarticular infection 1 (50), SSTI 1 (50)	Aminopenicillin and inhibitor 1 (50) Anti-staphylococcal penicillin 1 (50) Quinolone 2 (100)	0 (0)
Shah et al., 2013 [30]	1	Male	64	Heart, BSI	Carbapenem Vancomycin	1
Pandey et al., 2024 [31]	3	1 female, 2 males	72, 80, 83	Heart 3 (100), BSI 3 (100)	Vancomycin 3 (100) Daptomycin 1 (33.3) Linezolid 1 (33.3) Aminoglycoside 2 (66.7) Rifampicin 3 (100)	0 (0)

BSI: bloodstream infection; LRTI: lower respiratory tract infection; NR: not reported; SSTI: skin and soft tissue infection.

**Table 2:** Characteristics of patients with *Kytococcus* species infection.

Characteristic	All Patients (*n* = 30) *	Bacteremia (*n* = 20) *#	Infective Endocarditis (*n* = 11) *	Lower Respiratory Tract Infection (*n* = 7) *	Bone and Joint Infection (*n* = 5) *
Age, years, median (IQR)	59.5 (42.3–71)	51.5 (21.3–70.8)	64 (49–73)	52 (43–68)	71 (47.5–79.5)
Male gender, *n* (%)	17 (56.7)	12 (60)	7 (63.6)	4 (57.1)	1 (20)
Predisposing factors					
Prosthetic cardiac valve, *n* (%)	11 (36.7)	11 (55)	11 (100)	0 (0)	0 (0)
Immunosuppression, *n* (%)	8 (26.7)	6 (30)	0 (0)	6 (85.7)	0 (0)
Hematological malignancy, *n* (%)	8 (26.7)	6 (30)	0 (0)	6 (85.7)	0 (0)
Recent surgery, *n* (%)	2 (6.7)	1 (5)	1 (9.1)	0 (0)	1 (20)
Rheumatic fever, *n* (%)	1 (3.3)	1 (5)	1 (9.1)	0 (0)	0 (0)
Previous infective endocarditis, *n* (%)	1 (3.3)	1 (5)	1 (9.1)	0 (0)	0 (0)
End-stage renal disease on dialysis, *n* (%)	1 (3.3)	0 (0)	0 (0)	0 (0)	0 (0)
Polymicrobial infection, *n* (%)	2 (6.7)	1 (5)	0 (0)	1 (14.3)	0 (0)
Clinical characteristics					
Fever, *n* (%)	19/29 (65.5)	16 (80)	8 (72.7)	6 (85.7)	1 (20)
Sepsis, *n* (%)	11/29 (37.9)	11 (55)	5 (45.5)	2 (28.6)	0 (0)
Microbiology					
*K. schroeteri*, *n* (%)	25 (83.3)	18 (90)	11 (100)	6 (85.7)	4 (80)
*K. sedentarius*, *n* (%)	4 (13.3)	1 (5)	0 (0)	1 (14.3)	1 (20)
*Kytococcus* spp., *n* (%)	1 (3.3)	1 (5)	0 (0)	0 (0)	0 (0)
Treatment					
Vancomycin, *n* (%)	20/28 (71.4)	17/19 (89.5)	10 (90.9)	4/5 (80)	0 (0)
Rifampicin, *n* (%)	11/28 (39.3)	9/19 (47.4)	8 (72.7)	0/5 (0)	1 (20)
Aminoglycoside, *n* (%)	7/28 (25)	7/19 (36.8)	7 (63.6)	0/5 (0)	0 (0)
Quinolone, *n* (%)	6/28 (21.4)	2/19 (10.5)	1 (9.1)	1/5 (20)	3 (60)
Carbapenem, *n* (%)	4/28 (14.3)	4/19 (21.1)	1 (9.1)	1/5 (20)	0 (0)
Aminopenicillin and inhibitor, *n* (%)	2/28 (7.1)	0/19 (0)	0 (0)	0/5 (0)	1 (20)
Cephalosporin, *n* (%)	2/28 (7.1)	2/19 (10.5)	0 (0)	2/5 (40)	0 (0)
Daptomycin, *n* (%)	2/28 (7.1)	1/19 (5.3)	1 (9.1)	0/5 (0)	1 (20)
Linezolid, *n* (%)	2/28 (7.1)	2/19 (10.5)	1 (9.1)	1/5 (20)	0 (0)
Tetracycline, *n* (%)	1/28 (3.6)	0/19 (0)	0 (0)	0/5 (0)	1 (20)
Trimethoprim-sulfamethoxazole, *n* (%)	1/28 (3.6)	1/19 (5.3)	0 (0)	1/5 (20)	0 (0)
Anti-staphylococcal penicillin, *n* (%)	1/28 (3.6)	0/19 (0)	0 (0)	0/5 (0)	0 (0)
Teicoplanin, *n* (%)	1/28 (3.6)	1/19 (5.3)	1 (9.1)	0/5 (0)	0 (0)
Surgical management, *n* (%)	16 (53.3)	10 (50)	8 (72.7)	0 (0)	4 (80)
Treatment duration, weeks, median (IQR)	6 (5–6)	6 (5.5–6.5)	6 (6–7)	NA	6 (5–7)
Outcomes					
Deaths due to infection, *n* (%)	8 (26.7)	6 (30)	1 (9.1)	6 (85.7)	0 (0)
Deaths overall, n (%)	9 (30)	7 (35)	1 (9.1)	6 (85.7)	0 (0)

IQR: interquartile range; NA: not applicable. * Data are among the number of patients mentioned on top unless otherwise described; # Cases of bacteremia include cases with infective endocarditis, five cases of lower respiratory tract infections, and one case of central nervous system infection.

**Table 3:** Antimicrobial resistance rates of *Kytococcus* spp.

Antimicrobial Agent	Number of Patients *	Resistance (%)
Macrolides	10/10	100
Penicillin	16/17	94.1
Oxacillin	13/14	92.9
Cephalosporin	10/11	90.9
Clindamycin	5/8	62.5
TMP-SMX	2/6	33.3
Aminoglycosides	2/17	11.8
Quinolones	1/11	9.1
Rifampicin	1/16	6.3
Vancomycin	1/23	4.3
Carbapenem	0/6	0
Tetracyclines	0/11	0
Linezolid	0/10	0
Daptomycin	0/5	0

TMP-SMX: trimethoprim-sulfamethoxazole; * Data show number of strains being resistant to the respective antimicrobial divided by the number of strains with corresponding data. The rest of the cases did not have available data regarding antimicrobial resistance for the respective antimicrobial agent.

**Table 4:** Characteristics of patients with *Kytococcus* species infection with regard to outcome.

Characteristic	Survived (*n* = 21) *	Died (*n* = 9) *	*p*-Value
Age, years, median (IQR)	66 (44–72.5)	52 (28.5–66)	0.3091
Male gender, *n* (%)	10 (47.6)	7 (77.8)	0.2293
Predisposing factors			
Prosthetic cardiac valve, *n* (%)	10 (47.6)	1 (11.1)	0.1
Immunosuppression, *n* (%)	2 (9.5)	6 (66.7)	0.0031
Hematological malignancy, *n* (%)	2 (9.5)	6 (66.7)	0.0031
Recent surgery, *n* (%)	2 (9.5)	0 (0)	1
Rheumatic fever, *n* (%)	1 (4.8)	0 (0)	1
Previous infective endocarditis, *n* (%)	1 (4.8)	0 (0)	1
End-stage renal disease on dialysis, *n* (%)	1 (4.8)	0 (0)	1
Polymicrobial infection, *n* (%)	1 (4.8)	1 (11.1)	0.5172
Type of infection			
Bacteremia, *n* (%)	13 (61.9)	7 (77.8)	0.6749
Infective endocarditis, *n* (%)	10 (47.6)	1 (11.1)	0.1
Lower respiratory tract infection, *n* (%)	1 (4.8)	6 (66.7)	0.0009
Osteoarticular infection, *n* (%)	5 (23.8)	0 (0)	0.2860
Central nervous system infection, *n* (%)	1 (4.8)	0 (0)	1
Skin and soft tissue infection, *n* (%)	1 (4.8)	1 (11.1)	0.5172
Peritoneal dialysis peritonitis, *n* (%)	1 (4.8)	0 (0)	1
Vascular graft infection, *n* (%)	1 (4.8)	0 (0)	1
Clinical characteristics			
Fever, *n* (%)	11/20 (55)	8 (88.9)	0.1071
Sepsis, *n* (%)	6/20 (30)	5 (55.6)	0.2371
Microbiology			
*K. schroeteri*, *n* (%)	18 (85.7)	7 (77.8)	0.6220
*K. sedentarius*, *n* (%)	3 (14.3)	1 (11.1)	1
*Kytococcus* spp., *n* (%)	0 (0)	1 (11.1)	0.3
Treatment			
Vancomycin, *n* (%)	14 (66.7)	6/7 (85.7)	0.6334
Rifampicin, *n* (%)	11 (52.4)	0/7 (0)	0.0233
Aminoglycoside, *n* (%)	7 (33.3)	0/7 (0)	0.1414
Quinolone, *n* (%)	5 (23.8)	1/7 (14.3)	1
Carbapenem, *n* (%)	2 (9.5)	2/7 (28.6)	0.2530
Aminopenicillin and inhibitor, *n* (%)	2 (9.5)	0/7 (0)	1
Cephalosporin, *n* (%)	0 (0)	2/7 (28.6)	0.0556
Daptomycin, *n* (%)	2 (9.5)	0/7 (0)	1
Linezolid, *n* (%)	2 (9.5)	0/7 (0)	1
Tetracycline, *n* (%)	1 (4.8)	0/7 (0)	1
Trimethoprim-sulfamethoxazole, *n* (%)	1 (4.8)	0/7 (0)	1
Anti-staphylococcal penicillin, *n* (%)	1 (4.8)	0/7 (0)	1
Teicoplanin, *n* (%)	1 (4.8)	0/7 (0)	1
Surgical management, *n* (%)	16 (76.2)	0 (0)	0.0007

IQR: interquartile range; * Data are among the number of patients mentioned on top unless otherwise described.

**Table 5:** Logistic regression analysis of the overall mortality of patients with *Kytococcus* species infection.

Characteristic	Univariate Analysis *p*-Value	Multivariate Analysis *p*-Value	OR (95% CI)
Immunosuppression	0.0006	0.062	16 (0.875–292.726)
Lower respiratory tract infection	<0.0001	0.314	4 (0.269–59.415)

CI: confidence interval; OR: odds ratio.

## Data Availability

Not applicable.
